# DynamicVLN: Incorporating Dynamics into Vision-and-Language Navigation Scenarios

**DOI:** 10.3390/s25020364

**Published:** 2025-01-09

**Authors:** Yanjun Sun, Yue Qiu, Yoshimitsu Aoki

**Affiliations:** 1 Department of Electronics and Electrical Engineering, Faculty of Science and Technology, Keio University, 3-14-1, Hiyoshi, Kohoku-ku, Yokohama 223-8522, Japan; aoki@elec.keio.ac.jp; 2 National Institute of Advanced Industrial Science and Technology (AIST), 1-1-1 Umezono, Tsukuba 305-8560, Japan; qiu.yue@aist.go.jp

**Keywords:** vision-and-language navigation, dynamic change, decision-making

## Abstract

Traditional Vision-and-Language Navigation (VLN) tasks require an agent to navigate static environments using natural language instructions. However, real-world road conditions such as vehicle movements, traffic signal fluctuations, pedestrian activity, and weather variations are dynamic and continually changing. These factors significantly impact an agent’s decision-making ability, underscoring the limitations of current VLN models, which do not accurately reflect the complexities of real-world navigation. To bridge this gap, we propose a novel task called Dynamic Vision-and-Language Navigation (DynamicVLN), incorporating various dynamic scenarios to enhance the agent’s decision-making abilities and adaptability. By redefining the VLN task, we emphasize that a robust and generalizable agent should not rely solely on predefined instructions but must also demonstrate reasoning skills and adaptability to unforeseen events. Specifically, we have designed ten scenarios that simulate the challenges of dynamic navigation and developed a dedicated dataset of 11,261 instances using the CARLA simulator (ver.0.9.13) and large language model to provide realistic training conditions. Additionally, we introduce a baseline model that integrates advanced perception and decision-making modules, enabling effective navigation and interpretation of the complexities of dynamic road conditions. This model showcases the ability to follow natural language instructions while dynamically adapting to environmental cues. Our approach establishes a benchmark for developing agents capable of functioning in real-world, dynamic environments and extending beyond the limitations of static VLN tasks to more practical and versatile applications.

## 1. Introduction

The Vision-and-Language Navigation (VLN) [1] task requires an agent to be able to navigate in environments based on visual inputs and natural language instructions. This capability is crucial for various real-world applications, including household robots, caregiver assistance, navigation aids for visually impaired individuals, disaster area assessment, and delivery services. There is a strong societal demand for advancements in this technology, as it has the potential to transform everyday life and support critical operations. To support these applications, researchers have proposed a variety of VLN datasets that capture various navigation challenges, including Room-to-Room (R2R) [1] for indoor settings and Touchdown [2] for exploring outdoor urban navigation, as well as specialized datasets such as ALFRED [3] that introduce tasks that combine navigation with object interaction, further enriching the scope of VLN research.

However, these datasets were designed based on static environments, where the objects and layout remain unchanged, failing to encapsulate the unpredictable nature of the real world. The absence of dynamic elements such as moving cars, pedestrians, fluctuating traffic lights, and variable weather conditions limits the applicability of these datasets for preparing agents to navigate in environments that closely mimic daily scenarios.

**Figure 1:**
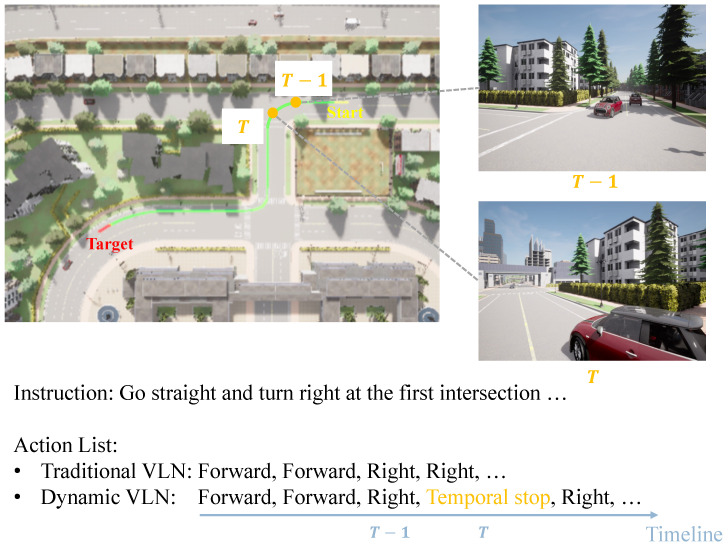
In traditional VLN tasks, agents predict actions based only on instructions, without accounting for real-time environmental changes. In Dynamic VLN tasks, however, agents must consider both instructions and dynamic elements, such as moving vehicles. For example, although the instruction here directs the agent to ‘turn right’, the agent must temporarily stop to yield to an oncoming car, adapting its actions to avoid a potential accident.

To address the limitations of traditional Vision-and-Language Navigation (VLN) in handling real-world dynamic scenarios, we introduce the Dynamic Vision-and-Language Navigation (DynamicVLN) task. DynamicVLN builds upon the traditional VLN framework by introducing a *temporal stop* action to handle the unpredictability of dynamic obstacles encountered during navigation. In traditional VLN, navigation is inherently a discrete, instruction-driven task, where the agent follows a predefined path strictly dictated by navigation instructions. The agent navigates step-by-step on a graph-based map, selecting the next adjacent node based on the given instruction. DynamicVLN extends this framework by incorporating temporal stop points, allowing agents to pause momentarily when encountering dynamic elements before continuing along the predefined path. Specifically, DynamicVLN is structured around four key scenarios: vehicles, pedestrians, traffic signals, and weather conditions. Each of these elements introduces variability that requires agents to dynamically adjust their behavior, closely mirroring humans’ challenges in everyday navigation. In response to these elements, agents may encounter numerous situations where they must decide whether to perform a *temporal stop* or to continue according to instructions. As shown in Figure 1, at timestep *T*, while the instruction indicates “turn right”, the agent must instead briefly stop to avoid a collision with an oncoming vehicle, exemplifying the need for adaptive decision-making in dynamic environments. This scenario highlights the challenges addressed by DynamicVLN, which requires agents to respond to their surroundings while adhering to navigation instructions dynamically.

Scenarios like this are among the 10 types of dynamic variations incorporated into the DynamicVLN dataset, designed to evaluate agents’ adaptability across diverse real-world challenges. Specific scenarios requiring a *temporal stop* are systematically detailed in Section 3. For example, in vehicle-related scenarios, an agent must navigate sudden stops by cars or adjust its path in response to vehicles merging into its lane. Pedestrian scenarios test the agent’s ability to safely navigate around individuals crossing the street unexpectedly or moving in unpredictable patterns. In scenarios involving traffic signals, agents are required to interpret changes in traffic lights, making split-second decisions that ensure compliance with traffic laws while progressing toward their goal. Weather scenarios introduce visual and physical challenges, such as reduced visibility due to fog or altered road conditions caused by rain or snow, requiring agents to modify their navigation strategies to maintain safety and efficiency. We constructed DynamicVLN using the CARLA simulator [4] and automatically generated instructions with GPT-4 [5], resulting in a dataset of 11,261 unique navigation instances. Each of the 10 dynamic scenarios—vehicles, pedestrians, traffic signals, and weather conditions—is divided between cases requiring a *temporal stop* and those that do not. This balanced design ensures comprehensive coverage of dynamic change detection, providing a robust foundation for training and evaluating navigation systems in real-world-like environments.

Along with the DynamicVLN task, we introduced DynaNav, a baseline model designed to address its distinct challenges effectively. At the core of DynaNav is a dynamic detection module, which recognizes dynamic elements within the environment. This module enables DynaNav to discern when to execute a ‘temporal stop’ or proceed, ensuring effective and adaptive navigation in dynamic scenarios. In summary, the contribution of our work is four-fold:

- We introduce Dynamic Vision-and-Language Navigation (DynamicVLN), a novel task that incorporates dynamic real-world scenarios such as moving vehicles, pedestrians, fluctuating traffic signals, and varying weather conditions, addressing the limitations of traditional static VLN tasks.
- We constructed the DynamicVLN dataset, comprising 11,261 navigation instances across ten dynamic scenarios. Data collection was automated using the CARLA simulator, and captions were generated automatically by GPT-4, ensuring both realism and diversity.
- We propose DynaNav, a baseline model equipped with a Dynamic Detection Module, enabling agents to recognize dynamic elements and make context-aware decisions, such as when to execute a ‘temporal stop’.

## 2. Related Works

### 2.1. Vision-and-Language Navigation Dataset

Vision-and-Language Navigation (VLN) tasks require agents to navigate environments using natural language instructions. Existing VLN datasets encompass a variety of scenarios. For indoor navigation, the Room-to-Room (R2R) dataset [1], based on Matterport3D [6], serves as a foundational benchmark. It has been extended by Room-Across-Room (RxR) [7] and XL-R2R [8], which include multilingual instructions. These datasets are designed for simple and structured navigation tasks. For outdoor navigation, the Touchdown dataset [2], utilizing Google Street View (https://developers.google.com/maps/documentation/streetview, accessed on 15 September 2024), provides a benchmark for navigating complex urban environments. Similarly, StreetLearn [9], Retouchdown [10], StreetNav [11], Talk2Nav [12], map2seq [13], and VLN-VIDEO [14] focus on urban navigation tasks, with VLN-VIDEO augmenting navigation performance using driving videos. In terms of object interaction, the ALFRED dataset [3], built upon AI2-THOR 2.0 [15], emphasizes complex tasks requiring agents to interact with objects while navigating indoor environments. For aerial navigation, the AerialVLN dataset [16], based on the AirSim (version 1.2.0) [17], introduces challenges that require agents to interpret instructions and navigate elevated perspectives. Some datasets focus on task-specific navigation; for instance, CARLA-NAV [18] explores the grounding of navigable regions corresponding to textual descriptions, while DOROTHIE [19] highlights dialogue-based navigation in dynamic environments. However, these datasets primarily address static or predictable dynamic cues and lack realistic, sudden changes commonly encountered in real-world traffic scenarios.

In contrast, DynamicVLN addresses this limitation by introducing sudden, unpredictable events—such as abrupt vehicle stops, unexpected pedestrian crossings, and changing traffic signals—into the navigation process. It preserves the discrete, instruction-driven structure of traditional VLN tasks, ensuring agents’ decision-making remains interpretable and consistent with existing VLN frameworks. Additionally, the integration of temporal stop actions allows agents to effectively respond to real-world dynamic challenges without deviating from predefined paths. (The key differences and contributions of DynamicVLN compared to other datasets are systematically summarized in Table 1).

### 2.2. Approach for Vision-and-Language Navigation

With the introduction of numerous Vision-and-Language Navigation (VLN) benchmarks, researchers have developed various methods to enhance navigation performance. Initially, many approaches employed sequence-to-sequence (seq2seq) models, integrating images and instructions into pre-trained models [21,22,23], which have great understanding for images and instructions, then utilizing cross-modal attention mechanisms for action prediction [1,24]. Transformer-based models have further advanced VLN performance. ORIST [25] introduced object-level and word-level inputs to learn fine-grained relationships across textual and visual modalities, enabling more precise decision-making. VLN-BERT [26] extended this approach by developing a recurrent vision-and-language BERT, which incorporates historical states to enhance sequential decision-making in navigation tasks. SOTA [27] proposed a scene- and object-aware transformer, emphasizing context-specific understanding by focusing on relevant objects and environmental details. Given the challenges of environmental understanding in VLN tasks, researchers have also incorporated fine-grained attention mechanisms to improve agents’ comprehension of their surroundings. For example, previous work [28,29] leveraged landmarks along the route to divide the navigation path into smaller segments, using these landmarks as references to enhance navigation performance by providing clear intermediate goals. Recently, LLM-based approaches have emerged as a promising direction in VLN. For instance, VELMA [30] employs large language models to generate natural language explanations, helping agents reason about their navigation steps. VirtuWander [31] introduced a method for augmenting VLN tasks with detailed descriptions generated by LLMs, improving interpretability and task success. MapGPT [32] integrates GPT-based language generation to facilitate map-based navigation, showcasing the potential of LLMs to handle complex, multimodal navigation tasks.

### 2.3. Large Language Model for Dataset Generation

Recent advancements in large language models (LLMs) have significantly enhanced data annotation processes. Traditional methods often involve manual labeling, which is time-consuming and costly. Applications of synthetic data generation have garnered significant attention due to their remarkable capability to understand and generate human-like text based on input prompts and a few examples [33]. Early works utilized LLMs primarily for generating textual data, particularly in the natural language processing (NLP) field, enabling tasks such as text classification [34], summarization [35], and translation with minimal human intervention [36]. Building on this foundation, LLMs have also been applied in multimodal contexts. For instance, LLaVA [37] demonstrated how language-only models like GPT-4 could generate multimodal instruction-following data by combining vision encoders with LLMs to create datasets for visual understanding tasks. Moreover, LLMs have been leveraged to augment existing datasets to boost task performance. AttrPrompt [38] utilized LLMs to enhance attribute-based reasoning tasks, while other studies employed LLMs to expand datasets with diverse, high-quality examples [39]. Beyond general-purpose datasets, there has been a growing focus on using LLMs to generate domain-specific datasets [40,41].

In this work, we design a multistage workflow to generate high-quality navigation instructions for DynamicVLN automatically. To address the hallucination issues, we utilize two LLMs in our workflow. One LLM generates the instructions, while the other supervises and corrects the outputs, ensuring the realism, diversity, and accuracy of the dataset.

## 3. DynamicVLN Dataset

### 3.1. Task Definition

DynamicVLN is a task that challenges an agent to navigate through an environment based on natural language instructions $X=\{x_{1},x_{2},\ldots ,x_{l}\}$ while dynamically adapting to changes within that environment to reach a specified target location. The instructions consist of a sequence of *l* word tokens, each represented by $x_{i}$. The environment is structured as an undirected graph, with nodes v∈V representing specific locations connected by labeled edges (v,u)∈E, where *u* denotes an adjacent location to *v*. Each node is linked to an RGB image, providing visual context, and edges denote possible navigation paths with heading angles $\alpha_{\left(v,u\right)}$ between images.

At any given time *t*, the agent’s state is $s_{t}\in S,s_{t}=\left(v_{t},\alpha_{\left(v_{t-1},v_{t}\right)}\right)$, incorporating the current panoramic view $v_{t}$ and the heading angle $\alpha_{\left(v_{t-1},v_{t}\right)}$ from the previous state to the current one. To navigate, the agent performs actions from the set $a_{t}\in \left\{\mathrm{forward},\mathrm{left},\mathrm{right},\mathrm{temporalstop},\mathrm{stop}\right\}$, where the temporal stop action is introduced to allow brief pauses in response to dynamic events such as moving pedestrians or vehicles.

The objective in DynamicVLN is for the agent to generate a sequence of state-action pairs $\langle \left(s_{1},a_{1}\right),\left(s_{2},a_{2}\right),\ldots ,\left(s_{n},a_{n}\right)\rangle$, culminating in a $a_{n}=\mathrm{STOP}$ action that indicates the goal location has been reached, as defined by the instructions. Including the *temporal stop* action enhances the agent’s ability to navigate more effectively by adapting to real-time changes in the environment, thus making DynamicVLN a more realistic and challenging task that mirrors the complexities of navigating dynamic, real-world scenarios.

### 3.2. Scenario Design

We designed four dynamic element types to realistically simulate complex navigation environments: vehicles, pedestrians, traffic signals, and weather. Each element introduces specific trigger conditions that require agents to make adaptive decisions while maintaining alignment with the discrete, instruction-driven structure of VLN tasks.

**Figure 2:**
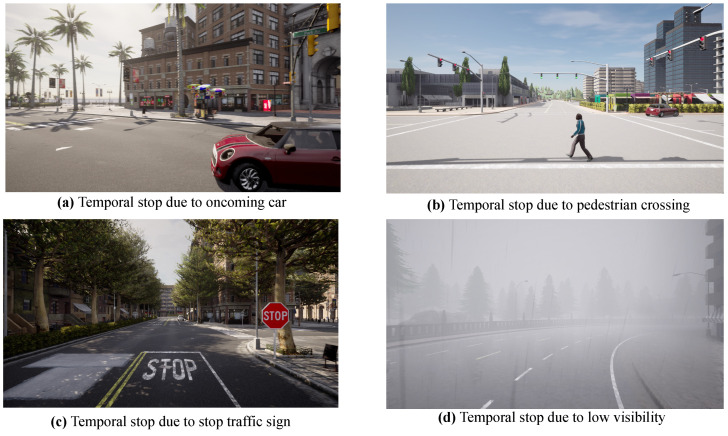
Example of temporal stop under each dynamic element type setting.

For instance, these scenarios often necessitate a temporal stop when agents encounter immediate risks or obstructions—such as yielding to cross-traffic, halting for a pedestrian crossing, or stopping in response to a traffic signal change. These trigger conditions and their corresponding decision logic are systematically summarized in Table 2, which highlights how each dynamic element aligns with temporal stop or continue actions based on specific environmental cues. Figure 2 shows examples in DynamicVLN when the vehicle needs to temporarily stop at timestep *T* during driving for each type of dynamic element.

### 3.3. Dataset Collection

We designed driving scenarios using the open source CARLA simulator [4] to simulate realistic driving conditions and collect images and driving data on vehicle navigation. Specifically, we utilized CARLA’s pre-defined town maps (Town 1, 2, 3, 4, 5, 6, and 10 HD) as our environment. In each map, we defined waypoints spaced 5 m apart to serve as navigable points along the routes. We generated random traffic flows, including vehicles and pedestrians, to simulate real-world traffic conditions and create dynamic scenarios. Initially, we select each defined waypoint as a start waypoint to create static routes. Each path spans 30–50 waypoints and includes at least one turning intersection, mimicking the design of traditional VLN datasets. These static paths serve as the baseline for our dataset. We introduced dynamic elements to construct dynamic routes based on these static routes, simulating real-world driving conditions with unpredictable events.

Specifically, we defined a vehicle automatically driving on the routes and set up a camera sensor on the car to capture images during driving behavior. We collected the actions (forward, right, left, temporal stop, stop) and landmarks for each waypoint on the routes. We collected the actions according to the route yaw changes and vehicle speed. The landmarks are mainly about traffic signs.

### 3.4. Instruction Generation

**Figure 3:**
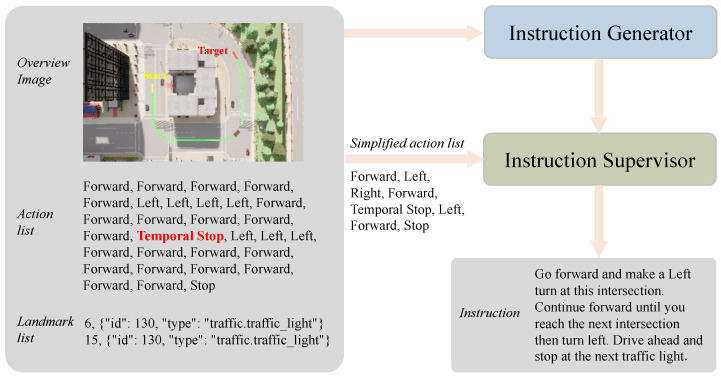
Pipeline of instruction generation for DynamicVLN, and this example shows an emergency scenario with oncoming cars.

Based on the data collected from CARLA, we proposed a pipeline to generate high-quality navigation instructions using GPT-4o automatically. In this work, we aimed to train the agent to adapt to sudden events, so we did not add the temporal stop action in the instructions. This pipeline employs two LLM agents: an **Instruction Generator** and an **Instruction Supervisor**. As shown in Figure 3, the pipeline begins by feeding the route overview, the sequence of actions at each waypoint, and the landmarks encountered along the route into the **Instruction Generator**. The generator produces an initial navigation instruction formatted similarly to traditional VLN instructions. Next, the generated instruction and a simplified action list are passed to the **Instruction Supervisor**. The supervisor evaluates the alignment between the simplified action list and the generated instruction, checking for missing actions, incorrect sequencing, or unnecessary additions. Based on its analysis, the supervisor refines the instruction to ensure accuracy and consistency with the action list. This iterative process ensures that the final instruction is coherent, is aligned with the route’s actions, and accurately reflects the dynamic elements of the environment.

**Figure 4:**
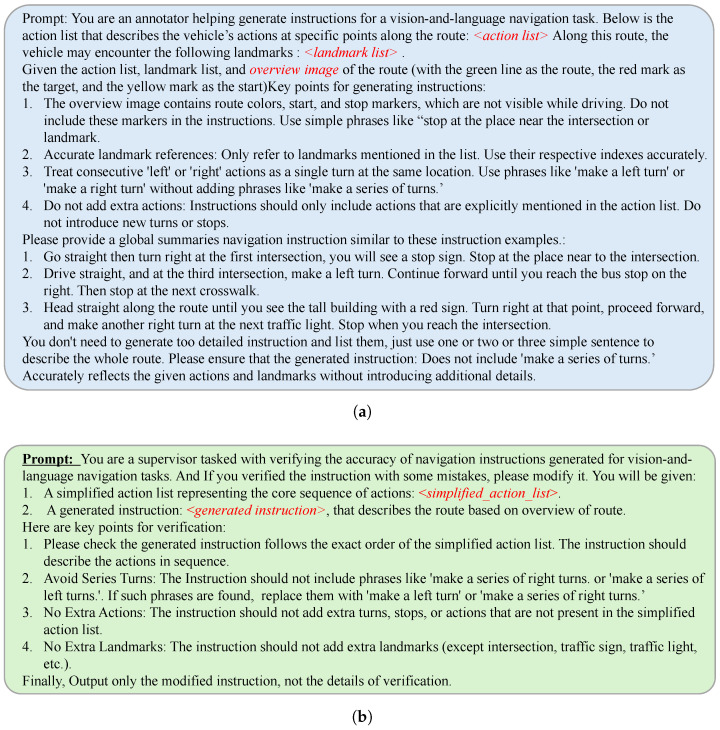
Prompts used in the instruction generation pipeline. The (**a**) shows the generation process, while the (**b**) illustrates the supervision and refinement process. (**a**) The Instruction Generator processes the route overview, action list, and landmarks to generate an initial navigation instruction. (**b**) The Instruction Supervisor refines the initial instruction by ensuring alignment with the simplified action list and correcting any discrepancies.

Specifically, in Figure 4, we provide the prompts used by the **Instruction Generator** and the Instruction Supervisor during the instruction creation process. The **Instruction Generator** receives structured input detailing the route overview, actions, and landmarks, generating an initial instruction. The Instruction Supervisor takes the simplified action list and the initial instruction as input and performs a consistency check, identifying necessary corrections before refining the final instruction.

### 3.5. Data Statistics

**Figure 5:**
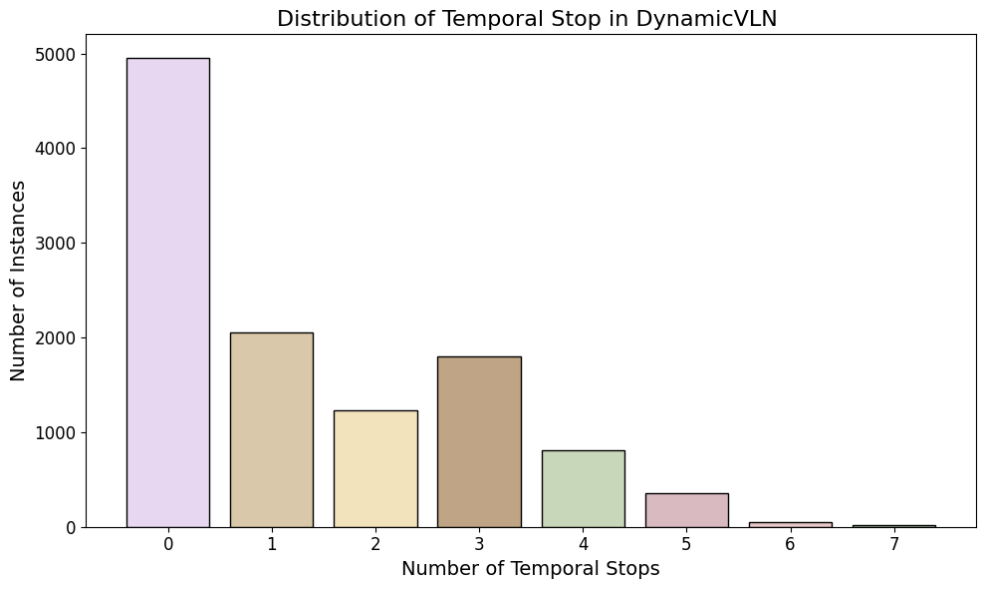
Distribution of routes based on the number of temporal stops. This figure shows the frequency of routes containing different numbers of temporal stops, reflecting the complexity and variability of dynamic scenarios in the collected dataset.

Following the pipeline introduced above, we collected a total of 11,261 routes. Among these, 2786 routes are associated with dynamic elements of vehicles, 1680 with pedestrian activities, 3370 with traffic conditions, and 601 with weather-related scenarios. These represent various dynamic factors encountered during navigation. However, the occurrence of a dynamic factor does not necessarily require the vehicle to stop temporally. In many cases, vehicles may need to stop at multiple points along the route before they can proceed, rather than at a single temporal stop. To better understand these dynamics, we analyzed the distribution of temporal stops within these routes. Each dynamic route involves between two and seven temporal stop actions, reflecting the complexity of real-world scenarios where vehicles must repeatedly adjust to changing conditions. Furthermore, each dynamic instance has 2–7 temporal stops. Figure 5 illustrates the distribution of routes based on the number of temporal stops they contain, providing insight into the frequency and variation of such actions across all collected routes.

## 4. Method

**Figure 6:**
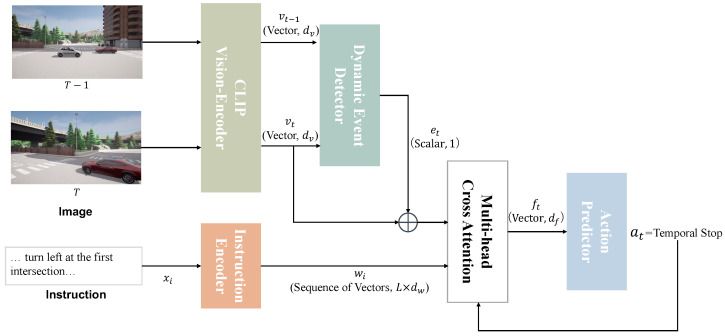
Overview for proposed model DynaVLN.

In this section, we introduce the DynaVLN model, designed to detect dynamic events and improve navigation performance in dynamic environments, building upon traditional VLN models. As illustrated in Figure 6, DynaVLN consists of four key components: the *Image Encoder*, *Instruction Encoder*, *Dynamic Event Detector*, and *Action Predictor*. At each decoding timestep, DynaVLN computes a visual representation of the agent’s current and previous states in the environment, integrating previously predicted actions, instruction features, and dynamic event detection results to predict the next action.

### 4.1. Model Details

**Image Encoder.** At each timestep *t*, the agent captures an image view of its surroundings. The visual representation of the current agent position is computed by extracting features from the panorama using a pre-trained CLIP Vision Encoder [42]. This step provides a robust and compact visual embedding $v_{t}$ for navigating and detecting changes during the driving process.

**Instruction Encoder.** The instruction encoder processes the natural language navigation instructions $x=\{x_{1},x_{2},\ldots ,x_{L}\}$, where *L* denotes the number of tokens in the instruction sequence. Each token $x_{i}$ is embedded and encoded using a bidirectional LSTM [43]:

(1) $$\begin{array}{cc} {\hat{x}}_{i} & =\mathrm{embedding}(x_{i}) \end{array}$$

(2) $$\begin{array}{cc} \left(w_{1},w_{2},\ldots ,w_{L}\right),z_{L}^{w} & =\text{Bi-LSTM}({\hat{x}}_{1},{\hat{x}}_{2},\ldots ,{\hat{x}}_{L}), \end{array}$$

where $w_{i}$ represents the hidden representation of token $x_{i}$, and $z_{L}^{w}$ is the final cell state of the LSTM. These outputs capture both local (token-level) and global (sequence-level) contextual information from the navigation instructions.

**Dynamic Event Detector.** To enable the agent to handle unforeseen dynamic events without explicit instructions intelligently, we incorporate a *Dynamic Event Detector* (DED) and propose an *Attention Modulation Mechanism* that dynamically adjusts the model’s focus between visual and language features based on the detected event. This mechanism allows the model to autonomously decide actions, including *temporal stop*, to address sudden changes in the environment.

At each timestep *t*, the DED processes the current visual feature $v_{t}$ and the previous visual feature $v_{t-1}$ to compute the event signal $e_{t}$, representing the likelihood of a dynamic event. Formally, the event signal is defined as follows:

(3) $$e_{t}=\sigma \left(\mathrm{MLP}\left(\left[v_{t}-v_{t-1}\right]\right)\right)$$

where σ(·) is the sigmoid activation, and MLP denotes a lightweight multi-layer perceptron. $e_{t}\in \left[0,1\right]$ indicates the intensity of the detected dynamic event.

The event signal $e_{t}$ is then used to modulate the attention mechanism, dynamically altering the interaction between the visual feature $v_{t}$ and the instruction feature sequence $\left\{w_{1},\ldots ,w_{L}\right\}$. The modulation affects both the Query ($q_{t}$) and the Key/Value representations ($k_{t},v_{k}$) as follows:

(4) $$\begin{array}{cc} q_{t} & ={\mathrm{Linear}}_{q}\left(\left[v_{t};e_{t}\right]\right) \end{array}$$

(5) $$\begin{array}{cc} k_{t},v_{k} & ={\mathrm{Linear}}_{k}\left(\left[w_{1},\ldots ,w_{L}\right]\right)\cdot \left(1-e_{t}\right),\;{\mathrm{Linear}}_{v}\left(\left[w_{1},\ldots ,w_{L}\right]\right)\cdot \left(1-e_{t}\right) \end{array}$$

where $\left[v_{t};e_{t}\right]$ denotes the concatenation of the visual feature and the event signal. The Key and Value representations are scaled by $\left(1-e_{t}\right)$, reducing the influence of language instructions when a significant dynamic event is detected ($e_{t}\to 1$).

The attention output $f_{t}$ is computed via the scaled dot-product attention mechanism:

(6) $$\mathrm{Attention}\left(q_{t},k_{t},v_{k}\right)=\mathrm{softmax}\left(\frac{q_{t}k_{t}^{⊤}}{\sqrt{d_{k}}}\right)v_{k}$$

where $d_{k}$ is the dimensionality of the Key vectors. The resulting feature $f_{t}$ incorporates the adjusted contribution of visual and language features, dynamically weighted by the presence or absence of a detected event.

**Action Predictor.** Finally, the attention output $f_{t}$ is concatenated with the event signal $e_{t}$ and passed to the Action Predictor to generate the next action:

(7) $$a_{t}=\mathrm{ActionPredictor}\left(\left[f_{t};e_{t}\right],a_{t-1}\right)$$

where $a_{t}$ is the predicted action (e.g., *move forward*, *turn left*, or *stop*) and $a_{t-1}$ is the previous action.

### 4.2. Loss Function

To train the proposed model effectively, we design a loss function that jointly optimizes the performance of the *Dynamic Event Detector* (DED) and the *Action Predictor*.

**Dynamic Event Detector Loss.** The DED outputs an event signal $e_{t}\in \left[0,1\right]$, representing the probability of a dynamic event occurring at timestep *t*. The ground truth event label $y_{t}\in \left\{0,1\right\}$ indicates whether a dynamic event is present. The detection loss is formulated as a binary cross-entropy loss:

(8) $$L_{\mathrm{DED}}=-\frac{1}{N}\sum_{t=1}^{N}\left[y_{t}\log\left(e_{t}\right)+\left(1-y_{t}\right)\log\left(1-e_{t}\right)\right],$$

where *N* is the total number of samples. This loss ensures that the DED module learns to predict event probabilities that align with the ground truth labels.

**Action Prediction Loss.** The *Action Predictor* generates the probability distribution over possible actions $\left\{a_{1},a_{2},\ldots ,a_{C}\right\}$, where *C* is the number of action classes (e.g., *move forward*, *turn left*, *stop*). The ground truth action label is denoted as $a_{t}^{\mathrm{true}}\in \left\{1,\ldots ,C\right\}$. We use a categorical cross-entropy loss to optimize the predicted action distribution:

(9) $$L_{\mathrm{Action}}=-\frac{1}{N}\sum_{t=1}^{N}\sum_{i=1}^{C}1\left[a_{t}^{\mathrm{true}}=i\right]\log P\left(a_{t}=i|f_{t},e_{t},a_{t-1}\right),$$

where $P(a_{t}=i|f_{t},e_{t},a_{t-1})$ is the predicted probability of action *i*, and 1[·] is the indicator function.

**Joint Loss.** To jointly optimize both components of the model, we combine the above losses into a single objective:

(10) $$L=\lambda_{1}L_{\mathrm{DED}}+\lambda_{2}L_{\mathrm{Action}},$$

where $\lambda_{1}$ and $\lambda_{2}$ are balancing weights that control the relative importance of the two loss terms.

## 5. Experiments

This section presents extensive experiments on our DynamicVLN dataset to evaluate our DynaVLN models’ performance on the outdoor VLN task and emergency accident adaptation.

### 5.1. Implementation Details

**Data Processing.** We adopted a clustering-based approach to group spatially proximate waypoints into unified nodes to construct a node-based navigation graph for each town map for Vision-and-Language Navigation (VLN) tasks. Specifically, all waypoints extracted from the routes, including those used during navigation, were collected and clustered based on their spatial proximity. For clustering, we utilized the DBSCAN algorithm [44], which is effective for identifying arbitrarily shaped clusters without requiring a predefined number of clusters. Each waypoint was treated as a three-dimensional point (x,y,z) in Cartesian coordinates, and waypoints within a distance threshold (ϵ=0.5 m) were grouped into the same cluster. After clustering, each cluster was assigned a unique node ID. The navigation graph was then constructed by treating each cluster as a node and connecting nodes adjacent to the same route.

**Model Details.** The proposed framework and baseline models were implemented using PyTorch (version 2.0.1) (https://pytorch.org/, accessed on 1 October 2024). We employed a pre-trained CLIP vision encoder with a ViT backbone to extract visual features with a size of 512 from images at each waypoint. Navigation instructions were tokenized into byte pair encodings (BPE) using a vocabulary size of 2000 tokens. The instruction tokens were lower-cased and embedded into vectors of size 256. For the loss function, we balanced the objectives of navigation performance and dynamic event detection using weights $\lambda_{1}=0.5$ and $\lambda_{2}=1.0$. This prioritization ensures the agent achieves reliable navigation while accurately detecting dynamic events. In scenarios where ground truth event labels $y_{t}$ are unavailable, pseudo-labels were generated heuristically. These heuristics included abrupt changes in the visual scene (e.g., the appearance of obstacles) or significant deviations in the agent’s planned actions (e.g., an unexpected stop or turn). This allows the model to handle dynamic events in complex environments adaptively. Since each town map is unique, it was necessary to construct a separate navigation graph for each town and conduct training individually for each map. However, in this section, we only present experimental results using the Town05 map. Town05 was selected because it contains the largest number of routes, with a total of 2611 routes. These routes were randomly split into training, development, and test sets in a 7:2:1 ratio, resulting in 1828 routes for training, 522 for development, and 261 for testing.

### 5.2. Baseline Models

**ORAR** [45] is a VLN model proposed for outdoor VLN tasks. This model uses an LSTM to encode the instruction text and an LSTM to decode the multimodal features and predict the following action. We selected ORAR because it is a model without collision detection and only uses whole images and instruction features for decision-making. By comparing our results with ORAR, we demonstrated that collision detection benefits adaptability to unforeseen events.

### 5.3. Metrics

The following metrics are used to evaluate VLN performance: (1) Task Completion (TC): the accuracy of navigating to the correct location. The correct location is defined as the exact goal panorama or one of its neighboring panoramas. (2) Shortest-Path Distance (SPD) [46]: the mean distance between the agent’s final position and the goal position in the environment graph. (3) Success Weighted by Edit Distance (SED): the normalized Levenshtein edit distance [47] between the predicted path and the ground truth path, with points only awarded for successful paths. (4) Coverage Weighted by Length Score (CLS) [48]: a measurement of the fidelity of the agent’s path to the ground truth path. (5) Normalized Dynamic Time Warping (nDTW) [49]: the minimized cumulative distance between the predicted path and the ground truth path. (6) Success Weighted Dynamic Time Warping (SDTW): the nDTW value where the summation is only over successful navigation.

### 5.4. Quantitative Results

We provide a comprehensive evaluation of the experimental results to assess DynaVLN’s performance in comparison to the baseline ORAR across various metrics. These results highlight the proposed model’s strengths and limitations, particularly in handling dynamic navigation environments. The experimental results are summarized in Table 3.

DynaVLN demonstrates higher TC, indicating its ability to navigate more effectively and reach target destinations more reliably. In terms of SPD, DynaVLN maintains a closer adherence to the target trajectory, reflecting its capability to navigate with greater precision. For SED, DynaVLN achieves better alignment with the ground truth action sequences, highlighting its accuracy in predicting correct actions during navigation. Conversely, ORAR achieves a higher CLS, suggesting stronger coverage of the navigation path but potentially less responsiveness to dynamic changes. Regarding temporal alignment, ORAR performs better in static environments, as indicated by its nDTW score. However, DynaVLN excels in dynamic scenarios, with a significantly higher sDTW score, showcasing its ability to adapt and succeed in environments with dynamic changes. Overall, these results suggest that while ORAR is well suited for structured and static environments, DynaVLN offers superior adaptability and robustness in dynamic and complex real-world navigation tasks.

## 6. Conclusions

In this paper, we addressed the challenge of navigating dynamic environments by introducing the Dynamic Vision-and-Language Navigation (DynamicVLN) task. Our work recognizes the limitations of traditional VLN tasks, which largely focus on static environments, and proposes a more realistic dataset that incorporates the unpredictability of real-world conditions.

Looking ahead, we plan to refine and perfect the DynamicVLN task. We will focus on developing the DynaNav model to meet the unique challenges presented by dynamic elements. DynaNav will incorporate advanced real-time decision-making techniques, leveraging visual and linguistic inputs to navigate effectively and safely.

## Figures and Tables

**Table 1:** Comparison of various Vision-and-Language Navigation datasets highlighting environment type, data source, presence of dynamic elements, use of automatic annotation, and primary task focus (the highlighted color in the table shows our DynamicVLN).

Dataset	Environment	Data Source	Dynamic Elements	Automatic Annotation	Emergent Adaptation	Complex Navigation Conditions
Room-to-Room [1]	indoor	Matterport3D	✗	✗	✗	Structured, static
Room-Across-Room [7]	indoor	Matterport3D	✗	✗	✗	Structured, static
VLN-CE [20]	indoor	Matterport3D	✓	✗	✗	Continuous navigation
ALFRED [3]	indoor	AI2-THOR 2.0	✓	✗	✗	Object interactions
Touchdown [2]	outdoor	Google Street View	✗	✗	✗	Urban navigation
map2seq [13]	outdoor	Google Street View	✗	✓	✗	Urban navigation
AerialVLN [16]	outdoor	AirSim	✓	✗	✗	Aerial navigation
CARLA-NAV [18]	outdoor	CARLA	✓	✗	✗	Grounded navigation
DOROTHIE [19]	outdoor	CARLA	✓	✗	✗	Dialogue-based navigation
VLN-VIDEO [14]	outdoor	Google Street View	✗	✓	✗	Urban navigation
DynamicVLN (our)	outdoor	CARLA	✓	✓	✓	Emergent adaptation

**Table 2:** Dynamic elements in DynamicVLN are abstracted into higher-level trigger conditions and corresponding decision logic, maintaining the discrete, instruction-driven nature of VLN tasks. Specific scenarios illustrate how agents respond to each condition through temporal stop or continuation decisions.

Dynamic Element	Trigger Condition	Decision Logic	Including Scenario
Vehicle	Obstacle appears in agent’s path	Temporal stop	Vehicle stops abruptly ahead
	Potential collision risk detected	Temporal stop or not	Vehicle approaches from side lane
	Priority vehicle detected	Temporal stop	Ambulance or fire engine approaches
	Lane change detected	Temporal stop or not	Vehicle changes lanes abruptly
Pedestrian	Pedestrian enters agent’s intended path	Temporal stop	Pedestrian crosses at crosswalk
	Sudden pedestrian movement detected	Temporal stop	Child runs across road
Traffic Condition	Change in traffic signal state	Temporal stop or not	Traffic light turns red
	Regulatory signs encountered	Temporal stop or not	Stop sign detected
Weather	Visibility reduced due to weather conditions	Temporal stop	Heavy fog or rain obscures view
	Road condition compromised	Temporal stop	Slippery road detected

**Table 3:** Quantitative results comparing **ORAR** and **DynaVLN** on navigation performance metrics. Higher TC and CLS scores indicate better trajectory completion and coverage length, respectively. Lower SPD, SED, nDTW, and sDTW scores indicate better alignment with ground truth trajectories. The quantity of each metric is defined as follows: TC is measured as a percentage (%), SPD represents graph distance, SED corresponds to edit distance, CLS measures coverage length in graph distance, and both nDTW and sDTW represent dynamic time warping scores. The up-arrow means higher is better, and the down-arrow means lower is better.

Method	TC ↑	SPD ↓	SED ↑	CLS ↑	nDTW ↑	sDTW ↑
**ORAR**	1.65	24.02	1.08	16.35	4.15	1.05
**DynaVLN**	2.74	23.65	1.41	15.07	3.26	2.10

## Data Availability

The data supporting the findings of this study are available from the corresponding author upon reasonable request. Additionally, a subset of our dataset is publicly available on GitHub at https://github.com/AuderySun/DynamicVLN, accessed on 10 November 2024. For further inquiries or access to the full dataset, please contact the corresponding author.

## Abbreviations

The following abbreviations are used in this manuscript:

VLN	Vision-and-Language Navigation
LLM	Large Language Model
GPT	Generative Pre-Trained Transformers
LD	Linear Dichroism
